# Hybrid parameters for fluid identification using an enhanced quantum neural network in a tight reservoir

**DOI:** 10.1038/s41598-023-50455-z

**Published:** 2024-01-11

**Authors:** Dejiang Luo, Yuan Liang, Yuanjun Yang, Xingyue Wang

**Affiliations:** 1 College of Mathematics and Physics, Chengdu University of Technology, Chengdu, 610059 China; 2 Geomathematics Key Laboratory of Sichuan Province, Chengdu University of Technology, Chengdu, 610059 China

**Keywords:** Applied mathematics, Energy science and technology

## Abstract

This paper proposes a fluid classifier for a tight reservoir using a quantum neural network (QNN). It is difficult to identify the fluid in tight reservoirs, and the manual interpretation of logging data, which is an important means to identify the fluid properties, has the disadvantages of a low recognition rate and non-intelligence, and an intelligent algorithm can better identify the fluid. For tight reservoirs, the logging response characteristics of different fluid properties and the sensitivity and relevance of well log parameter and rock physics parameters to fluid identification are analyzed, and different sets of input parameters for fluid identification are constructed. On the basis of quantum neural networks, a new method for combining sample quantum state descriptions, sensitivity analysis of input parameters, and wavelet activation functions for optimization is proposed. The results of identifying the dry layer, gas layer, and gas–water co-layer in the tight reservoir in the Sichuan Basin of China show that different input parameters and activation functions affect recognition performance. The proposed quantum neural network based on hybrid parameters and a wavelet activation function has higher fluid identification accuracy than the original quantum neural network model, indicating that this method is effective and warrants promotion and application.

## Introduction

Tight oil and gas reservoirs, which are situated in most parts of the world, have become a key research topic among petroleum and natural gas engineers, and their exploration and development has affected the world’s energy supply and demand patterns. In China, tight oil and gas reservoirs have become a crucial resource through which the unconventional oil and gas industry can increase reserves and production, and are widely distributed in the following basins: Ordos Basin^1^, Sichuan Basin^2,3^, Tarim Basin^4^, and Qaidam Basin^5^. Tight reservoirs are characterized by low porosity, low permeability, and strong inhomogeneity, and fluid identification crucially facilitates the formulation and adjustment of field development plans. It is difficult to identify fluids based on traditional statistical methods and cross-plots^6–8^. Fluid identification in tight reservoirs is particularly crucial; thus, its speed, accuracy, and economic sustainability should be optimized.

Datasets for fluid identification comprise seismic data^9,10^ and well log data^11^; the latter facilitates the effective analysis of the lithology and physical properties of reservoirs, and promotes the identification of fluid properties^12,13^. The fluid response of tight reservoirs is not apparent; well log data are affected by various factors, and the fluid properties of reservoirs cannot be accurately identified using only the data of single logs or reservoir parameters. The rapid development of big data has enabled successful reservoir evaluation and interpretation through data-driven machine learning methods^14^; algorithms such as the classification committee machine^6,15^, clustering^16^, neural networks^17,18^, discriminant analysis^19^, decision tree^20^, random forest^21^, and support vector machine^22^ have become prevalent, and integrated discriminant methods that combine many of these techniques have been utilized in reservoir fluid identification. Fluid identification based on nuclear magnetic logging and a Formation MicroScanner Image (FMI) is costly^8,23^, and development wells are rarely logged; by contrast, conventional logging data are available and abundant; hence, they form the basis of the input parameters for most fluid identification models^6,17,20–22^.

The fluid types in tight reservoirs are complex, and the observation samples are linearly non-separable. Hence recognition models and model input parameters must be investigated; thus, new intelligent algorithms can be built. Quantum neural networks combine quantum computing with neural networks, and they apparently optimize the computational efficiency of neural networks^23,24^. Since Kak proposed quantum neural networks, scholars have proposed quantum-derived neural networks^25^, quantum dot neural networks^26^, quantum cellular neural networks^27^, quantum associative storage algorithms^28^, and the construction of neural networks through the utilization of quantum rotating gates and controlled non-gates^29^. Due to research developments, applications in prediction^30,31^, image recognition^32^, classification^33,34^, and fault diagnosis^35^ have advanced. Quantum neural networks are applicable to reservoir evaluation^36–38^; however, they have not been applied to fluid identification in tight reservoirs.

In recent years, due to the enhancement of computer hardware performance and the continuous optimization of algorithms, artificial neural networks (ANN) and machine learning (ML) have revolutionized various fields^39^. In the field of computer vision, deep learning models such as convolutional neural networks (CNN) have surpassed human performance in image recognition tasks^40,41^. In the field of natural language processing, models such as recurrent neural networks (RNN) and long short-term memory (LSTM) can understand and generate natural languages^42,43^. In the field of medicine, artificial neural networks and machine learning have been widely utilized in tasks such as disease diagnosis, drug discovery, and the optimization of therapeutic programs^44–46^. In the field of energy, artificial neural network (ANN) models have been built to predict various energy-related problems, such as wax deposition, building energy efficiency, and oil field recovery, and a large amount of experimental data has been utilized^47–49^. Moreover, artificial neural networks can be combined with machine learning to predict reservoir porosity and permeability, and the ANN model exhibits satisfactory performance in predicting reservoir permeability and porosity, providing a data-driven approach for oil exploration and extraction^50,51^.

Fluid identification represents a difficult production and scientific problem for oil and gas exploration in tight reservoirs. Therefore, we refer to the novel quantum computing results to construct a novel recognition model that can enhance recognition accuracy. This study constructs a quantum neural network method based on a wavelet function (QNNw), which can effectively process data with large ambiguity and linearly indistinguishable data, and it utilizes the following preprocessing methods: observation samples for tight reservoirs, vector parameterization to analyze the sensitivity and correlation of the input parameters, and a wavelet function to activate the hidden layer. Thus, the model is optimized. To analyze the influence of different QNNs on identification performance, the proposed method (QNNw) is compared with a quantum neural network method that is based on a sigmoid function (QNNs).

The remainder of this paper is structured as follows. Data acquisition and preparation are described in “Methods and model training/methodology” section, and the proposed QNN is explained. “Experimental investigations and discussion” section presents the results and discussion, and “Conclusions” section offers the conclusions.

## Methods and model training/methodology

### Fluid feature extraction

#### Well log and rock physics parameters

Tight reservoir evaluation refers to the detection and quantification of lithology and fluid types through the interpretation of wellbore and well log data^52^. The input parameters of fluid identification can be divided into two main categories: (1) Well log parameters, which include borehole diameter (CAL), gamma ray (GR), transverse velocity (S-wave velocity, SV), longitudinal velocity (P-wave velocity, PV), shallow lateral resistivity (LLS), compensated neutron log (CN), deep lateral resistivity (LLD), and acoustic log (AC); ${C}_{1}=\{{\text{CAL}},{\text{GR}},{\text{SV}},{\text{PV}},{\text{LLS}},{\text{CN}},{\text{LLD}},{\text{AC}}\}$. (2) Rock physics parameters, which are sensitive to reservoir fluids^53^, include Poisson’s ratio $\upnu$, bulk modulus ${\text{K}}$, shear modulus $\upmu$, Young’s modulus ${\text{E}}$, Lamé coefficient $\uplambda$, and longitudinal-to-transverse velocity ratio ${V}_{p}/{V}_{s}$, which can be obtained using seismic or logging data as follows:

Young’s modulus $${\text{E}}$$

$$E=\frac{\rho }{\Delta {ts}^{2}}\times \left[\frac{3\Delta {ts}^{2}-4\Delta {tp}^{2}}{\Delta {ts}^{2}-\Delta {tp}^{2}}\right].$$

Bulk modulus $${\text{K}}$$

$$K=\uprho \times \frac{3\Delta {ts}^{2}-4\Delta {tp}^{2}}{3\Delta {ts}^{2}\times\Delta {tp}^{2}}.$$

Shear modulus $$\upmu$$

$$\mu =\frac{\rho }{\Delta {ts}^{2}}.$$

Poisson’s ratio $$\upnu$$

$$v=\frac{0.5\Delta {ts}^{2}-\Delta {tp}^{2}}{\Delta {ts}^{2}-\Delta {tp}^{2}}.$$

Lamé coefficient λ

$$\lambda =\uprho \times \left(\frac{{\Delta ts}^{2}-2{\Delta tp}^{2}}{\Delta {ts}^{2}\times\Delta {tp}^{2}}\right),$$

where $\Delta ts$($\mathrm{\mu s}/{\text{ft}}$) denotes S-wave time-difference, $\Delta tp$ ($\mathrm{\mu s}/{\text{ft}}$) denotes P-wave time-difference, $\rho$ (g/cm^3^) denotes density,

The rock physics parameters are expressed using the following formula: ${C}_{2}=\{E,K,\upmu ,\upnu ,\uplambda , \lambda \times \rho , \mu \times \rho , {V}_{p}/{V}_{s}\}$.

When a tight reservoir contains natural gas, the general Lamé coefficient × density (λ × ρ), shear modulus × density (μ × ρ), Poisson’s ratio $v$, bulk modulus K, and $\lambda /\mu$ all exhibit different response characteristics. Goodway analyzed the sensitivity of rock physical parameters and gave an amplitude versus offset (AVO) analysis based on fluid factors such as λ × ρ, μ × ρ, and $\lambda /\mu$^54^. Thus, in addition to identifying clastic lithologies and fluids with various rock physics parameters, the hybrid parameters of various rock physics parameters can also be utilized; thus, lithologies and fluids can be identified.

#### Fluid sensitivity analysis

Fluid characterization is dependent on the reliability and exactness of well log or rock physics parameters correlation^55^. For tight reservoirs, the sensitivity of well log parameters and rock physics parameters is analyzed using the sensitivity factor ${{\text{S}}}_{i}$ between the observed values of different parameters; ${{\text{S}}}_{i}$ considers the relative distance using a vector norm, and is computed as

1 $${{\text{S}}}_{i}=\frac{\Vert x-y\Vert }{\Vert x+y\Vert } x,y\in {\upomega }^{d},{\omega }^{f} i=\mathrm{1,2},\dots ,d,$$

where $d$ denotes the number of parameters, $x$ denotes the value of the well log parameters and rock physics parameters of the dry layer in the tight reservoir, ${\upomega }^{d}\mathrm{ denotes}$ the set of parameters of the dry layer, $y$ denotes the value of the well log and rock physics parameters of the fluid layer corresponding to $x$, and ${\omega }^{f}$ denotes the set pertaining to the property parameters of the fluid layer.

Equation (1) quantifies the sensitivity of the same parameter for the dry and fluid layers; however, it negates the correlation between the parameters. Due to the presence of redundant information between parameters, modeling and solution become more difficult, and the effectiveness of fluid identification may be affected. Scatter plots are the simplest and most effective method of analyzing the correlation of input parameters^11^. To provide optimized input parameters for the tight reservoir fluid identification model, we analyze the correlation between two sets of well log and rock physics parameters.

### Quantum neural network

#### Architecture of quantum neural network

**Figure 1:**
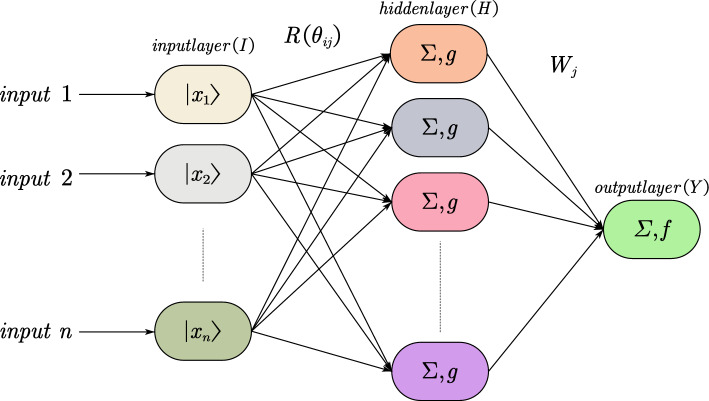
Quantum neural network structure.

Many methods of combining the quantum theory with biological neural networks exist; therefore, quantum neural network models can be implemented. We utilize Narayanan’s quantum neural network to construct a model for tight reservoir fluid identification^24,56^ that is similar in structure to a feedforward neural network (BPNN); however, the following exception can be observed: a quantum neuron is utilized as the hidden layer unit. In the implicit layer, a multilevel transfer function can adjust the quantum intervals during training. Figure 1 indicates that the quantum neural network comprises n input neurons, m hidden neurons, and one output neuron^24,56^.

Sample preprocessing method: Suppose there are N d-dimensional fluid observation samples $\left({x}_{i},{y}_{i}\right),{x}_{i}\in {R}^{d},{y}_{i}\in {R}^{1}$, where input data ${x}_{i}$ comprises corresponding output data ${y}_{i}$. The fluid identification parameters exhibit different magnitudes and large differences in sample values. We normalize d-dimensional fluid input samples ${\text{S}}=\{{{\text{s}}}_{1},{{\text{s}}}_{2},\dots ,{{\text{s}}}_{n}\}$ before inputting them to the QNN (Eq. 7) with values in the interval [0, 1]:

2 $${\mathrm{\varphi }}_{{\text{in}}}=\frac{{{\text{x}}}_{{\text{im}}}-{{\text{b}}}_{{\text{i}}}}{{{\text{a}}}_{{\text{i}}}-{{\text{b}}}_{{\text{i}}}},$$

$${{\text{b}}}_{i}={\text{max}}\,{x}_{i} \,{{\text{a}}}_{i}={\text{min}}\,{x}_{i} \,i=\mathrm{1,2},\dots ,m,$$

where $${{\text{a}}}_{i}={\text{min}}\,{x}_{i}, {{\text{b}}}_{i}={\text{max}}\,{x}_{i}, i=\mathrm{1,2},\cdots ,m$$.

##### First layer

The input layer comprises $n$ quantum neurons denoting the input parameters. The input ${x}_{i}$ of datasets $\left({x}_{i},{y}_{i}\right),{x}_{i}\in {R}^{d},{y}_{i}\in {R}^{1}$ is preprocessed in the interval $\left[0, 1\right]$, and encoded into the input state of the quantum neural network. The first layer converts them to quantum states ${\upphi }_{{\text{ij}}}$; thus, their phase values (Eq. 3) lie in the interval [0, π/2]^24^.

3 $${\upphi }_{{\text{ij}}}={\frac{\pi }{2}\mathrm{\varphi }}_{in}.$$

##### Second layer

The implicit layer receives the output value pertaining to the quantum neuron of the first layer, and the output of the* j*-th quantum neuron of the hidden layer is

4 $${h}_{j}={\text{sin}}\left(\frac{\uppi }{2}f\left({\mathrm{\alpha }}_{j}\right)-{\upbeta }_{j}\right),$$

where *f* denotes the activation function of the hidden content layer, and ${\mathrm{\alpha }}_{j}$ denotes the reversal parameter, which is determined for the initial value interval $\left[\mathrm{0,1}\right]$; the model learns and changes these parameters to attain the optimal value. ${\upbeta }_{j}$ denotes the quantum transition position that determines the shapes of quantum intervals, which represent adjustable parameters of the quantum neural network.

##### Third layer

This layer performs a weighted summation of the output ${h}_{j}$ of the hidden layer. The output of the input layer neurons can be obtained as

5 $${\widehat{y}}_{k}={\text{g}}\left({w}_{jk}{h}_{j}\right),$$

where ${\widehat{y}}_{k}$ denotes the actual output of the quantum neural network, ${\text{g}}$ denotes the chosen output function, and ${w}_{jk}$ denotes the connection power pertaining to the quantum neuron, which is situated in the hidden layer of the quantum neuron in the output layer.

Since the wavelet function exhibits satisfactory time–frequency localization characteristics and satisfactory resolution of the logging signal in both the time and frequency domains, it is utilized as the activation function (Eq. 6), herein denoted as QNNw, for the implicit layer of the quantum neural network; furthermore, the sigmoid function (Eq. 7), herein denoted as QNNs, is utilized for the output layer.

6 $${\text{g}}\left({\text{x}}\right)=\frac{{\text{cos}}1.75x}{{e}^{\frac{{x}^{2}}{2}}},$$

7 $${\text{f}}\left({\text{x}}\right)=\frac{1}{1+{{\text{e}}}^{-{\text{x}}}}.$$

#### Learning algorithm of quantum neural network

Suppose there are N *d*-dimensional fluid observation samples $\left({x}_{i},{y}_{i}\right),{x}_{i}\in {R}^{d},{y}_{i}\in {R}^{1}$, ${\text{y}}=({{\text{y}}}_{1},{{\text{y}}}_{2},\dots ,{{\text{y}}}_{N})$ is the actual output, $\widehat{{\text{y}}}=({\widehat{{\text{y}}}}_{1},{\widehat{{\text{y}}}}_{2},\dots ,{\widehat{{\text{y}}}}_{N})$ is the desired output, and the loss function is the mean squared error (MSE).

8 $${\text{MSE}}=\frac{1}{2}\sum \limits_{k=1}^{m}{\left({\widehat{y}}_{{\text{k}}}-{y}_{k}\right)}^{2}.$$

Minimizing the loss function is an optimization problem. The aim of learning is to minimize the root mean square error by modifying the network parameters. If we apply the gradient descent method to update the parameters ${\uptheta }_{ij}$, ${\mathrm{\alpha }}_{j}$, and ${w}_{jk}$ of the quantum neural network, the update formula for ${\uptheta }_{ij}$ can be described as

9 $${\uptheta }_{ij} \left({\text{t}}+1\right)={\uptheta }_{ij}\left(t\right)+\gamma\Delta {\uptheta }_{ij}\left(t\right),$$

10 $$\Delta {\uptheta }_{ij}=-\frac{\partial {\text{E}}}{\partial {\uptheta }_{ij}}={\sum }_{k=1}^{m}\left({\widehat{y}}_{k}-{y}_{k}\right){g}^{\mathrm{^{\prime}}}{\left({w}_{jk}{h}_{j}\right)w}_{jk}\cos\left(\frac{\uppi }{2}f\left({\mathrm{\alpha }}_{j}\right)-{\upbeta }_{j}\right)\frac{{{\text{T}}}_{{\text{ij}}}}{1+{{\text{S}}}_{{\text{j}}}^{2}},$$

11 $${T}_{ij}=\frac{{\text{cos}}\left({\varphi }_{in}+{\uptheta }_{ij}\right){S}_{j1}+{{\text{sin}}}^{2}\left({\varphi }_{in}+{\uptheta }_{ij}\right)}{{S}_{j1}^{2}},$$

12 $${{\text{S}}}_{{\text{j}}}=\frac{{\sum }_{{\text{i}}=1}^{{\text{n}}}{\text{sin}}\left({\mathrm{\varphi }}_{in}+{\uptheta }_{{\text{ij}}}\right)}{{\sum }_{{\text{i}}=1}^{{\text{n}}}{\text{cos}}\left({\mathrm{\varphi }}_{in}+{\uptheta }_{{\text{ij}}}\right)},$$

13 $${S}_{j1}={\sum }_{i=1}^{n}{\text{cos}}\left({\varphi }_{in}+{\uptheta }_{ij}\right).$$

The update formula for ${\mathrm{\alpha }}_{j}$ can be expressed as

14 $${\mathrm{\alpha }}_{j} \left({\text{t}}+1\right)={\mathrm{\alpha }}_{j}\left(t\right)+\gamma\Delta {\mathrm{\alpha }}_{j}\left(t\right),$$

15 $$\Delta {\mathrm{\alpha }}_{j}=-\frac{\partial {\text{E}}}{\partial {\mathrm{\alpha }}_{j}}=-\frac{\uppi }{2}{\sum }_{k=1}^{m}\left({\widehat{y}}_{k}-{y}_{k}\right){g}^{\mathrm{^{\prime}}}\left({w}_{jk}{h}_{j}\right){w}_{jk}{\text{cos}}\left(\frac{\pi }{2}f\left({\alpha }_{j}\right)-{\beta }_{j}\right){f}^{\mathrm{^{\prime}}}\left({\alpha }_{j}\right).$$

The update formula for ${w}_{jk}$ can be expressed as

16 $${w}_{jk} \left({\text{t}}+1\right)={w}_{jk}\left(t\right)+\gamma\Delta {w}_{jk}\left(t\right),$$

17 $$\Delta {w}_{jk}=-\frac{\partial {\text{E}}}{\partial {w}_{jk}}=-\left({\widehat{y}}_{k}-{y}_{k}\right){g}^{\mathrm{^{\prime}}}{\text{sin}}\left(\frac{\uppi }{2}f\left({\mathrm{\alpha }}_{j}\right)-{\upbeta }_{j}\right),$$

where $\gamma$ denotes the learning efficiency.

The QNN process for fluid identifications is expressed as follows:

- *Step 1* Select feature parameters, including well log parameter and rock physics parameters.
- *Step 2* Obtain the sample set, and divide it into training and test data.
- *Step 3* Perform sensitivity and correlation analyses to obtain the input parameters of fluid identification.
- *Step 4* Input training data, train the QNN, and determine the optimal parameters.
- *Step 5* Input training data pertaining to the optimal parameters, and train the WQNN.
- *Step 6* Perform a comparative analysis based on accuracy, recall, and loss.

## Experimental investigations and discussion

### Study area and fluid identification solution design

#### Study area and data

The study area is located in the Xujiahe Formation, Sichuan Basin, China, which is mainly located in Chengdu, Deyang, Mianyang, and Jiangyou in western Sichuan. The thickness of Xujiahe Formation is 400–700 m, with a maximum thickness of 1000 m near An County, which is adjacent to Longmen Mountain. The average porosity of the tight reservoirs is only 4.2%, and the differences between the logging curves of gas- and non-gas-bearing sands, as well as those of sands with different gas abundances, are small. The tight gas reservoirs in the study area are divided into gas layer (GL), water-bearing gas layer (WBGL), gas-bearing water layer (GBWL), and dry layer (DL) formations. For layers in tight reservoirs, the quantization {DL, GBWL, WBGL, GL} is expressed as {0.15, 0.40, 0.65, 0.90}, whose values are all fractional. The transfer function of the output layer is set to a sigmoid activation function; thus, the actual and desired output values can be bounded in the interval $\left[0, 1\right]$.

#### Fluid identification scheme design

The number of samples pertaining to each layer varies for the tight reservoirs located in the study area. To realize more globally representative network training samples, they were randomly selected; thus, the fluid identification model was trained. 70% of the samples are utilized for training, and the data generated by the sampling method Bootstrap for cross-validation. 30% of the samples are utilized for testing. To enhance the reliability of the experimental comparison, different input parameters of the fluid recognition model were utilized, the QNN and WQNN network models were trained and tested 30 times with identical training and test samples, and the experimental results were averaged for utilization as the evaluation index of the network training results.

### Selection of model input parameters

**Figure 2:**
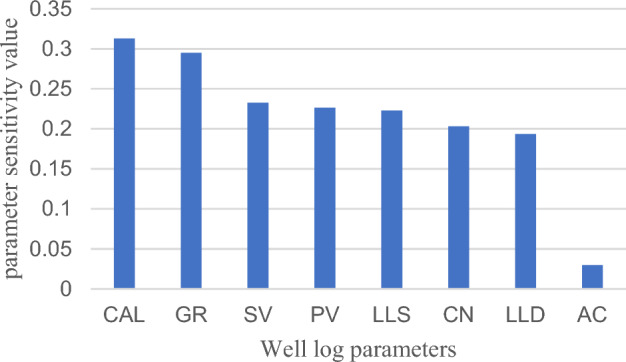
Well log parameter sensitivity analysis.

**Figure 3:**
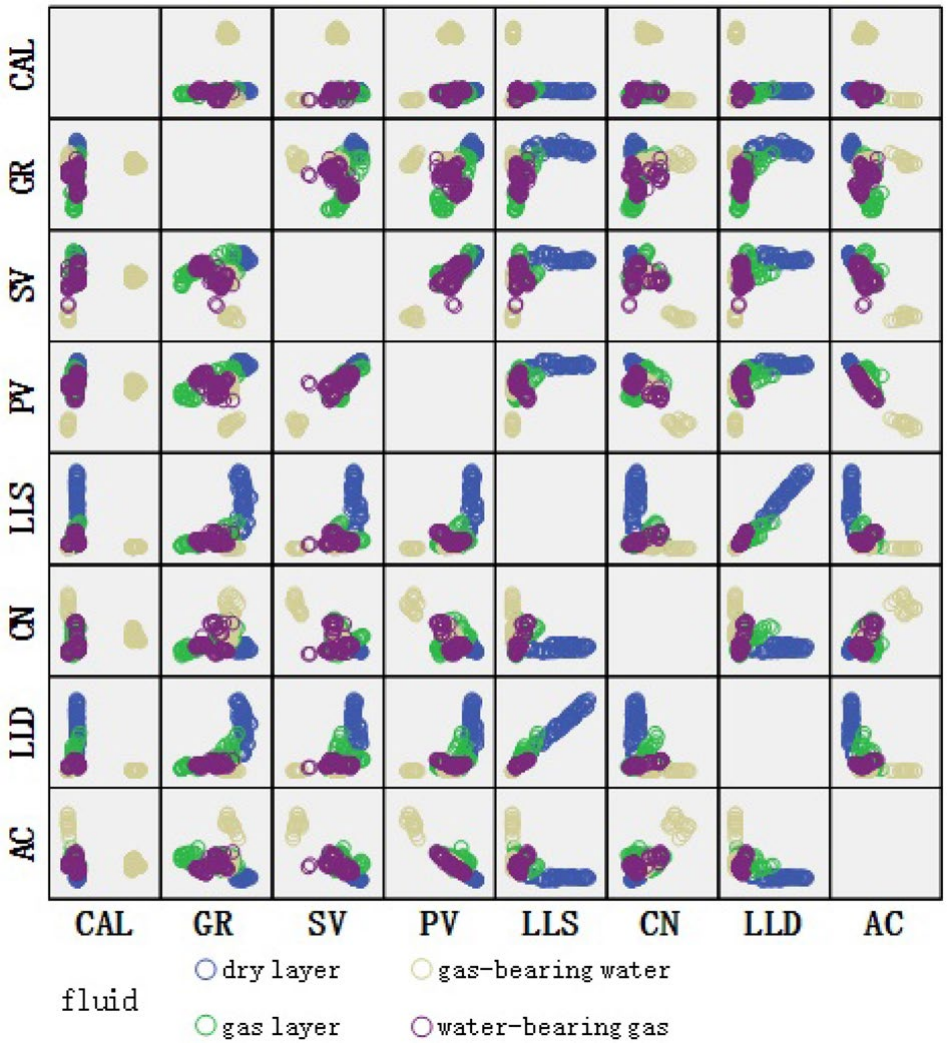
Well log parameter correlation analysis.

Input parameters for the fluid identification model were determined by sensitivity and correlation analyses. The sensitivity factor ${{\text{S}}}_{i}$ between the parameter values of the same well log parameter of the dry and fluid layers (Eq. 6) was calculated; thus, the fluid sensitivity parameters of the target reservoir were obtained (Fig. 2). According to the results pertaining to the sensitivity analysis of the input parameters (Fig. 1), the sensitivity values of each well log parameters were calculated using Eq. (6); with the exception of the parameter “AC” which has a sensitivity value of 0.03, the sensitivity factor ${{\text{S}}}_{i}$ of the other parameters CAL, GR, SWV, PWV, LLS, LLD, CN and LLD are all greater than 0.19, and the sensitivity values are all high They can be initially identified as fluid identification model input parameters. Further correlation analysis was performed on the initially selected sensitivity parameters. In the scatter plot depicted in Fig. 3, the well log parameters SV and PV exhibit apparent correlation, similar to LLS and LLD; therefore, only SV and LLS are retained as model input parameters. The sensitivity and correlation analyses create a scenario in which the well log parameters CAL, GR, SV, LLS, and CN are defined as the final input parameters 1 (IP1) for fluid identification.

**Figure 4:**
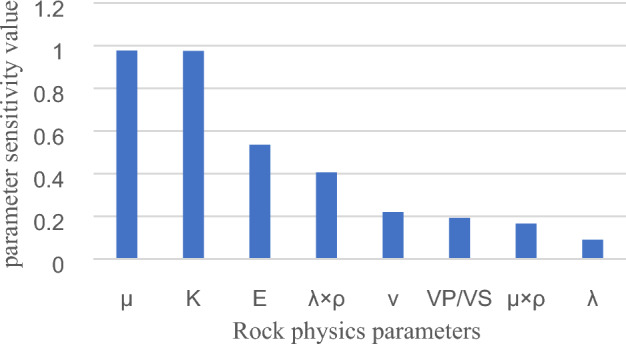
Rock physics parameters sensitivity analysis.

**Figure 5:**
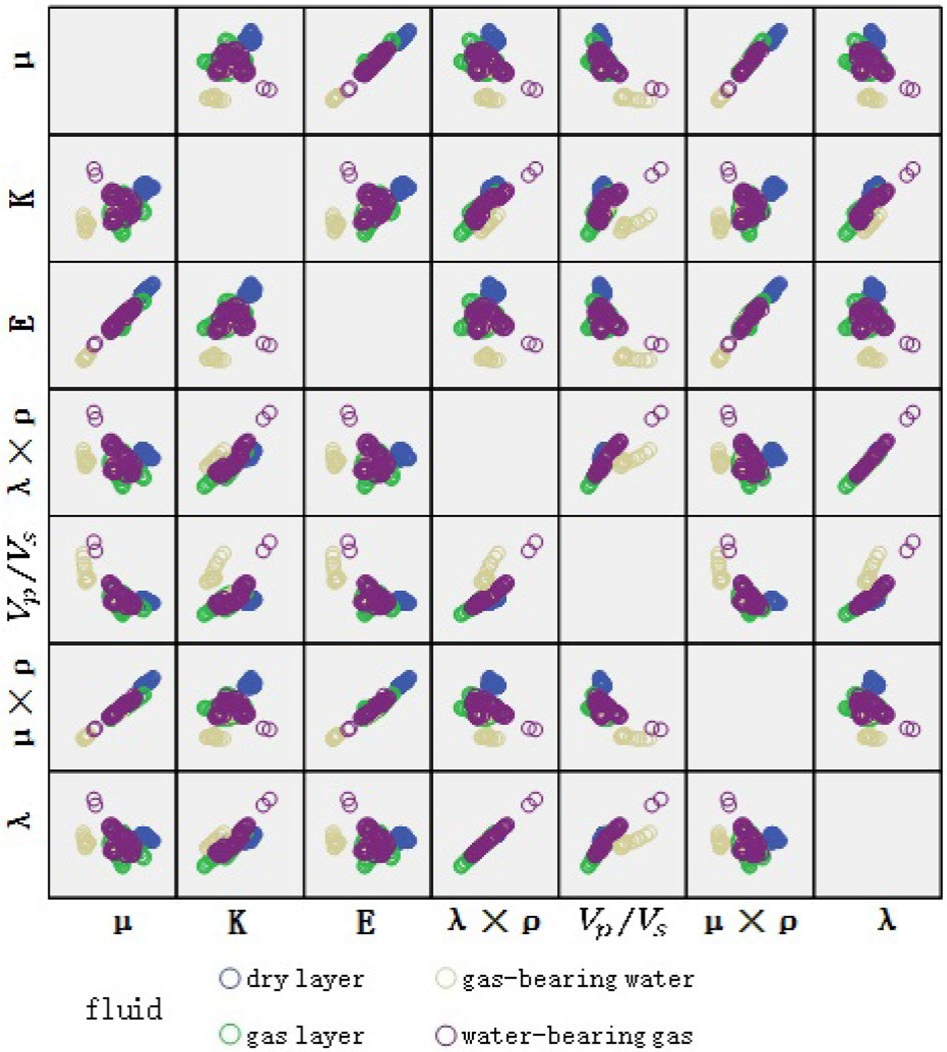
Rock physics parameters correlation analysis.

The rock physics parameters were obtained as per the preceding process. According to the results of the sensitivity analysis of the input parameters illustrated in Fig. 4, for the rock physics parameters (Fig. 2), $\mu$, K, E, λ × ρ, and $\upupsilon$ can be selected as model input parameters. In the scatter plot of Fig. 5, K and E exhibit apparent correlation, and only K is retained as a model input parameter. Through sensitivity and correlation analyses, the reservoir rock physics parameters $\mu$, K, E, λ × ρ, and $\upupsilon$ can be obtained as the final input parameters 2 (IP2) for fluid identification.

**Figure 6:**
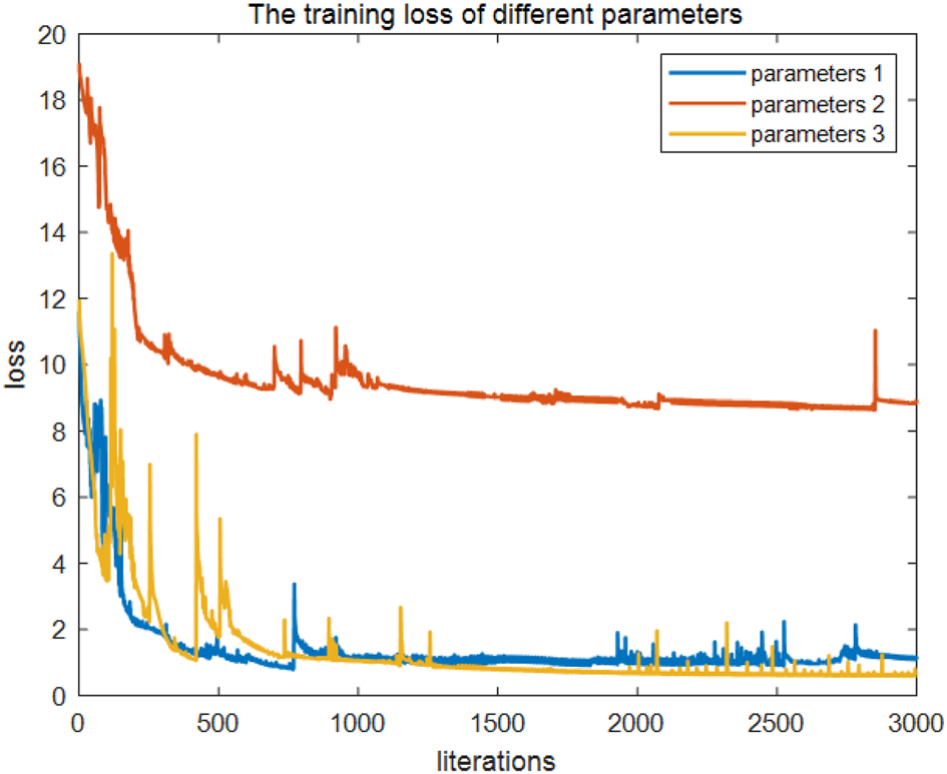
Loss function values of different parameters.

IP1 and IP2 can be combined to obtain the input parameters of the fluid recognition model, IP3 = ${S}_{1}=\{{\text{CAL}},{\text{GR}},{\text{SV}},{\text{PV}},{\text{LLS}},{\text{CN}},{\text{LLD}},{\text{AC}},E,K,\upmu ,\upnu ,\uplambda , \lambda \times \rho , \mu \times \rho , {V}_{p}/{V}_{s}\}$. The QNN model was trained with IP1, IP2, and IP3 (Fig. 6). Initially, the loss function values of all three inputs reduces quickly, and this decrease slows down when the number of iterations exceeds approximately 200. The loss function value of the rock physics parameters, an input parameter of the fluid identification model, is greater than those of IP1 and IP3. When the number of iterations approximates 1800, IP3 loss function value is significantly smaller than that of IP1; thus, for tight reservoir fluid identification, IP3 can be utilized as the final input parameter.

Table 1 depicts the range of input variable values for the proposed model. Table 1 indicates that the input variable values of four types of reservoirs, namely gas layer, dry layer, water-bearing gas layer, and gas-bearing water layer, are overlapped, which is a linear non-separation scenario; due to this phenomenon, the difficulty of classification is increased.

**Table 1:** Outputs and input variables in the tight gas reservoirs.

Input parameters		Category of reservoir			
		GL	WBGL	GBWL	DL
Input	CAL (inc)	6.0–7.6	5.3–19	5.4–7.6	6.8–7.5
	GR (Api)	23–82	67–82	38–76	79–96
	PV (m/s)	4400–5700	3200–5100	4300–5500	5500–6000
	SV (m/s)	2700–3900	1600–3200	2100–3500	3400–3900
	LLS (Ω m)	33–490	2.0–28	63–314	310–1500
	$\mu$(Mpa)	18–34	6–25	11–30	32–39
	K (Mpa)	14–38	16–34	21–51	37–44
	E (Mpa)	44–72	18–58	32–68	76–88
	λ × ρ	1.6–53	27–55	13–70	38–55
	$\upupsilon$	0.01–0.25	0.17–0.37	0.08–0.35	0.14–0.20
Output		0.15	0.40	0.65	0.90

**Figure 7:**
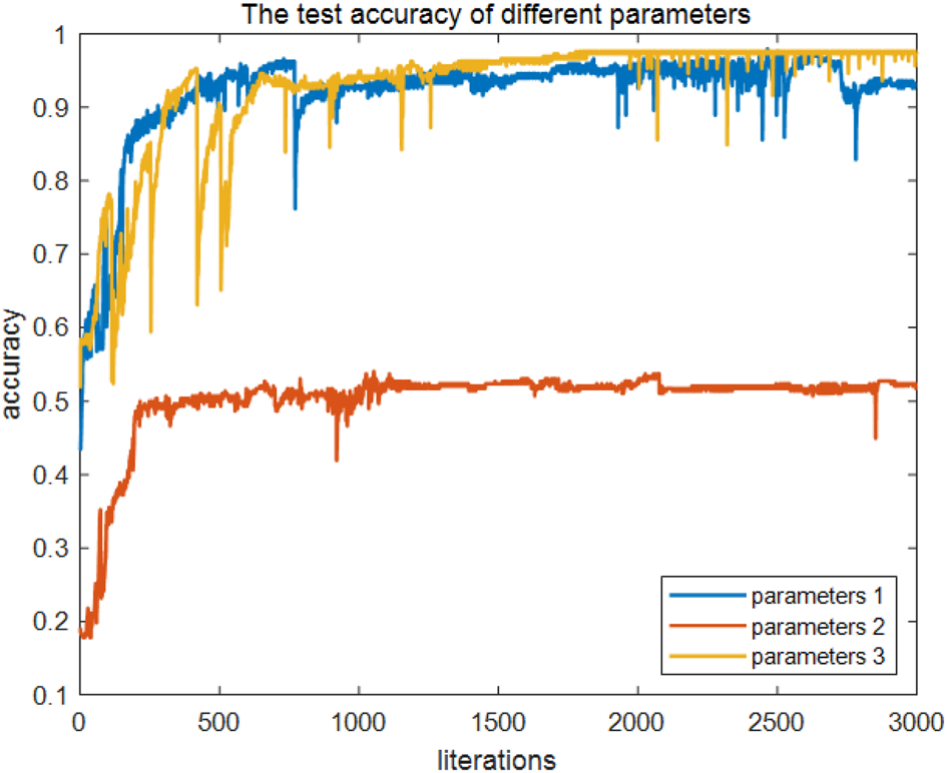
Test accuracy of different parameters.

**Figure 8:**
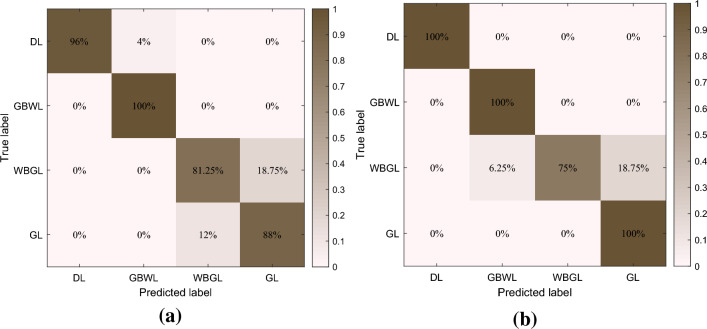
Recall of different parameters based on QNN. (**a**) Parameter 1 and (**b**) Parameter 3.

The trained fluid recognition model was utilized to classify the test samples, and the calculated classification accuracies that utilize IP1, IP2, and IP3 are illustrated in Fig. 7. When IP3 was selected, the classification accuracy was much lower than that of IP1 and IP3. The IP3 classification accuracy is generally more optimal than that of IP1 when the number of training sessions is > 1000. The normalized confusion matrix between the true values of the test samples and the classification results of the model when IP1 and IP3 are calculated without considering IP2 is depicted in Fig. 8. Regardless of the type of input parameters utilized, the QNN-based fluid recognition model performs effectively for DL and GBWL; for GL and WBGL, the model is less effective; and for GL and WBGL, IP3 is generally more optimal than IP1.

The effect of different input parameters on the fluid identification results is further analyzed using a confusion matrix, where diagonal elements indicate correctly identified reservoir types, with larger values implying higher classification accuracy. Other elements indicate misclassified reservoir types. Figure 8 indicates that when a sigmoid activation function and IP1 are utilized, the Recall is 96% for the dry layer; 100% for the gas-bearing water layer; 81.25% for the water-bearing gas layer, with 18.75% being identified as a gas-bearing water layer; and 88% for the gas layer, with 12% being identified as the water-bearing gas layer. When IP3 is, the recall is 100% for the dry layer and gas-bearing water layer; 75% for the water-bearing gas layer, 6.25% is identified as a gas-bearing water layer, 18.75 is identified as a gas layer; and the gas layer is 100%. In terms of recognition results, IP3 is better than IP1.

**Figure 9:**
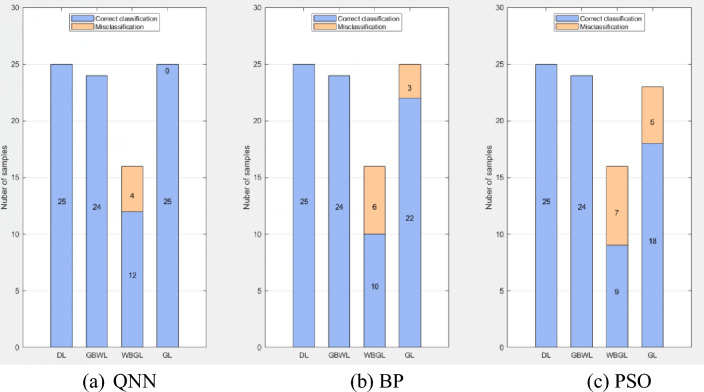
Histogram depicting the identification results of different methods.

If we consider the actual problem (i.e., fluid identification based on logging data), the recognition performance of different methods is depicted in Fig. 9, which is sufficient for the performance evaluation of the model in the test set. The blue bars indicate the number of correctly classified samples, and the red bars indicate the number of incorrectly classified samples. The three methods exhibit different recognition accuracies for the dry layer, water layer, gas layer, and gas–water layer; however, the overall recognition accuracy of the quantum neural network is higher.

### Comparison with QNN

We compare the performance of the QNN at the fluid recognition in which the sigmoid activation function (QNNs) and wavelet function (QNNw) are utilized, and we utilize a confusion matrix, where *TP* denotes the number of correctly classified positive samples, *TN* denotes the number of correctly classified negative samples, *FN* denotes the number of positive samples that are incorrectly classified as negative, and FP denotes the number of negative samples that are incorrectly classified as positive. Herein, the recognition effectiveness of QNN when different activation functions were utilized is evaluated using recall, precision (accuracy), and ${F}_{\beta }$, where recall denotes the percentage of reservoir fluid correctly classified as Class $\omega$; precision denotes the percentage of reservoir fluid that are exactly have Class $\omega$ between all those were classified as Class $\omega$; and correctness rate represents the ratio of the correct fluid type identified to the whole identified into that class. ${F}_{\beta }$ denotes a combined metric through which the single class accuracy and recall can be assessed, where $\upbeta =1$.

$${\text{Precision}}=\frac{TP}{TP+FN}\text{ or }{\text{Precision}}=\frac{TN}{TN+FP},$$

$${\text{Recall}}=\frac{TN}{TN+TP},$$

$${F}_{\beta }=\frac{(1+{\beta }^{2})\times precision\times recall}{{\beta }^{2}\times {\text{precision}}+{\text{recall}}}.$$

**Figure 10:**
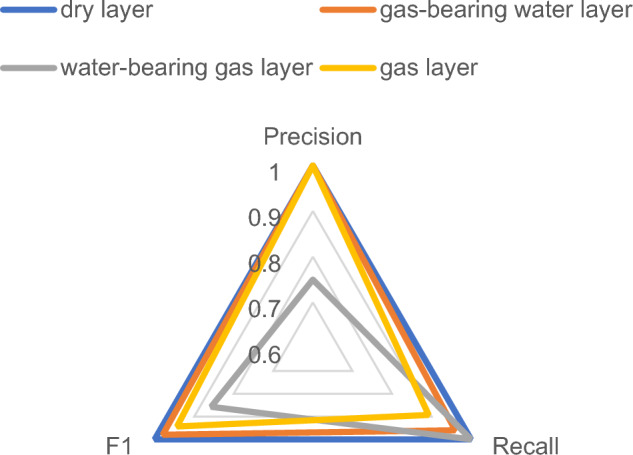
Recall of sigmoid activation function.

**Figure 11:**
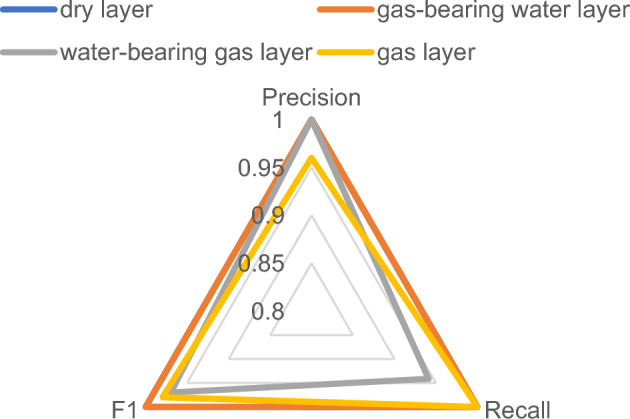
Recall of wavelet activation function.

Figures 10 and 11 indicate that the precision of the water-bearing gas layer increases from 0.75 to 1 when the wavelet function is utilized for QNN; however, the precision of the gas layer decreases slightly, from 1 to 0.96. With regard to recall, only the dry layer remains unchanged, whereas the recall of the water-bearing gas layer is enhanced from 0.96 to 1, and the recall increases from 0.8928 to 0.9213; however, the recall value for the water-bearing gas layer decreases slightly. The F1-score for the dry layer remains unchanged, whereas the F1 for the water-bearing gas layer, the gas-bearing water layer, and the gas layer all improve. The variation in the recall and precision in regard to F1 indicates that the fluid identification of tight reservoirs is enhanced when the quantum neural network utilizes wavelet functions. The dry layer exhibits the most optimal classification effect, possessing the highest F1-score, recall, and precision, and the gas-bearing water layer ranks second. The gas layer exhibits a higher precision and a slightly lower F1-score and recall, whereas the water-bearing gas layer exhibits a higher recall and a slightly worse recall for the other metrics.

**Figure 12:**
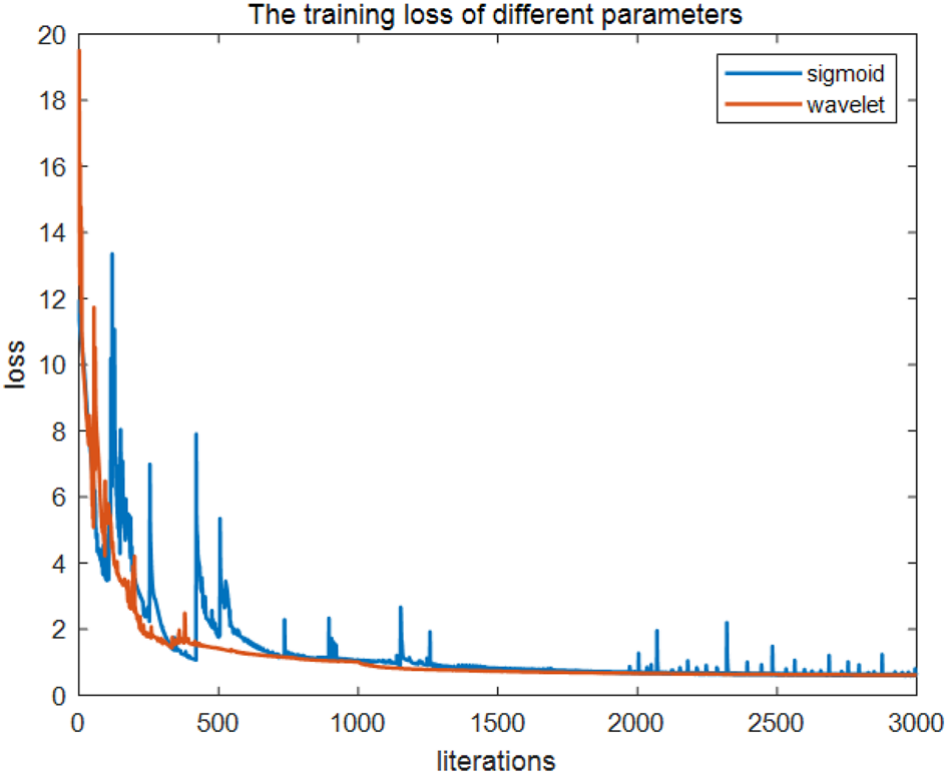
Training loss of different parameters.

**Figure 13:**
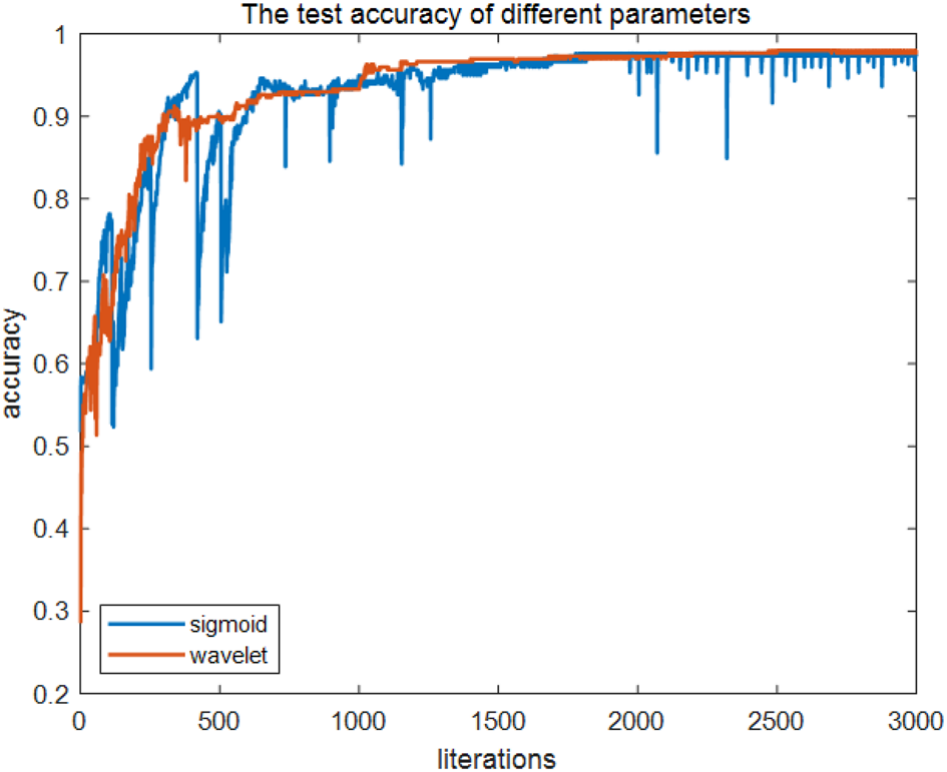
Accuracy of activation function on test set.

We utilized Eqs. (11) and (12) as activation functions for QNN; the activation functions are recorded as QNNs and QNNw, and the corresponding results are depicted in Figs. 12 and 13. Both QNNs and QNNw can achieve a satisfactory recognition effect; however, overall differences exist: the wavelet functions were somewhat more effective than the s-functions, and the smoothness observed on the plots was somewhat more satisfactory, without the violent vibration phenomenon, as was the case with sigmoid functions. Subsequently, accuracy was introduced; thus, the classification performance of QNNs and QNNw was compared as follows: the number of correctly classified reservoir fluids divided by the total of such fluids, which is expressed as

$${\text{Accuracy}}=\frac{TP+TN}{TP+TN+FN+FP}.$$

For the test dataset, when the number of training times exceeded 1000, the average recognition accuracy of both QNNs and QNNw was above 90%; however, the average recognition accuracy of QNNw was higher than that of QNNs, and the fluid recognition accuracy pertaining to the QNNw test dataset was 98%. The misidentified samples were concentrated in the gas layer, and some were identified as water-bearing gas layers.

Machine learning algorithms have been widely utilized in fluid identification, and can be compared and analyzed based on accuracy, recall, and ${F}_{\beta }$. To facilitate comparison, when considering only the accuracy, the accuracy of the quantum wavelet neural network proposed herein is higher than the accuracy of the decision tree classification model by 80.12^21^, the accuracy of the nearest neighbor algorithm classification model by 86.35, and the accuracy of the dynamic classification committee machine by 91.39^15^.

## Conclusions

The selection of input and model parameters for a quantum neural network model crucially impacts classification results. This study considered the aforementioned questions and attempted to enhance specific applications. By focusing on the problem of fluid identification in a tight reservoir, we analyzed the parameter selection of the identification model, performed sensitivity and correlation analyses, constructed an identification model and determined its parameters, and proposed a specific application of the algorithm. The main observations and the advantage of the proposed model are as follows:

1. A quantum neural network-based fluid identification scheme was investigated. Three sets of input indicators for the model were determined through sensitivity and correlation analyses, and the network was trained by applying known categories of reservoirs. The results indicated that quantum neural networks can be effectively utilized for fluid identification in tight reservoirs.
2. The performance of hybrid parameters was significantly more optimal than that of single-type parameters. Through the sensitivity and correlation analysis, well log parameter, rock physics parameters, and hybrid parameters were selected as different model inputs. The results indicated that the fluid identification effect is most optimal when mixed parameters are utilized.
3. Different activation functions of a quantum neural network affect the recognition result. Because a wavelet function exhibits satisfactory time–frequency localization characteristics, a Morlet wavelet function was utilized as an activation function in the hidden layer. Whether the input parameters were logging or mixed parameters, the recognition effect was more optimal than with a sigmoid function.

## Data Availability

The datasets generated during and/or analyzed during the current study are available from the corresponding author on reasonable request.
